# Search of anti-allodynic compounds from Plantaginis Semen, a crude drug ingredient of Kampo formula “Goshajinkigan”

**DOI:** 10.1007/s11418-019-01327-2

**Published:** 2019-06-12

**Authors:** Kazufumi Toume, Zhiyan Hou, Huanhuan Yu, Mitsuru Kato, Miki Maesaka, Yanjing Bai, Shiho Hanazawa, Yuewei Ge, Tsugunobu Andoh, Katsuko Komatsu

**Affiliations:** 1 Division of Pharmacognosy, Institute of Natural Medicine, University of Toyama, 2630 Sugitani, Toyama, Toyama, 930-0194 Japan; 2 Department of Applied Pharmacology, Graduate School of Medicine and Pharmaceutical Sciences, University of Toyama, 2630 Sugitani, Toyama, Toyama, 930-0194 Japan

**Keywords:** Plantaginis Semen, *Plantago asiatica*, Allodynia, Peripheral neuropathy, Iridoids, Goshajinkigan

## Abstract

Chemotherapy-induced peripheral neuropathy (CIPN) is one of the dose-limiting side effects of cancer chemotherapy. Although the control of CIPN is important, it is difficult to manage with currently available therapeutic drugs. Therefore, there is a need for novel therapeutic agents for treating CIPN. Goshajinkigan (GJG) is a Kampo formula composed of ten crude drugs. While GJG has been used for the treatment of CIPN, the active constituents of GJG and their underlying mechanisms of pharmacological effects are still unknown. Our previous study revealed that repetitive oral administration of the water extract of Plantaginis Semen, a crude drug ingredient of GJG, inhibited the mechanical allodynia induced by an intraperitoneal injection of paclitaxel in mice. To elucidate the active compounds of Plantaginis Semen, activity-guided separation of the water extract of Plantaginis Semen was performed. From the active fraction, four iridoids (**1**–**4**) were identified. Repetitive oral administration of aucubin (**1**) at 100 or 30 mg/kg and 100 mg/kg of the fraction **crude 3** [primarily comprised of pedicularis-lactone (**3**)], showed anti-allodynic activity, suggesting **1** and **3** could be some of the active compounds responsible for the anti-allodynic property of Plantaginis Semen and GJG. Our study establishes that oral administration of **1** has potent anti-allodynic effect in addition to the activity of intraperitoneally administered **1** reported previously. Identification of active anti-allodynic compounds found in Kampo formulations will support the development of novel therapies for the management of CIPN in cancer patients.

**Electronic supplementary material:**

The online version of this article (10.1007/s11418-019-01327-2) contains supplementary material, which is available to authorized users.

## Introduction

Chemotherapy is a widely used form of cancer therapy. Several chemotherapeutic agents including paclitaxel (PTX), oxaliplatin, vincristine, bortezomib, and others cause serious side effects such as vomiting and peripheral neuropathy. Chemotherapy-induced peripheral neuropathy (CIPN) is characterized by mechanical allodynia, paralysis, pain, tingling, and numbness, with a characteristic “stocking-and-glove” distribution of symptoms [1]. CIPN is one of the dose-limiting side effects in cancer chemotherapy and may persist even after the discontinuation of drug therapy. Even worse, CIPN leads to an impairment in patients’ quality of life (QOL) due to its effects on mobility and simple daily activities [2]. CIPN symptoms are difficult to manage using currently available therapeutic drugs. Furthermore, the underlying mechanism of CIPN is still not fully understood. Therefore, the discovery of new therapeutic agents to treat CIPN is an area of high priority.

Goshajinkigan in Japanese (GJG; 牛車腎気丸, Ji Sheng Shen Qi Wan in Chinese) [3], a Kampo (Japanese traditional medicine) formula composed of ten crude drugs [Rehmanniae Radix (地黄), Achyranthis Radix (牛膝), Corni Fructus (山茱萸), Dioscoreae Rhizoma (山薬), Plantaginis Semen (車前子), Alismatis Rhizoma (沢瀉), Poria (茯苓), Moutan Cortex (牡丹皮), Cinnamomi Cortex (桂皮), and Processi Aconiti Radix (附子)], has traditionally been used for the treatment of motor weakness of the lower back and legs, sensitivity to cold, pain, numbness, reduced micturition, nocturnal enuresis, edema, and lumbago [4], in addition to symptoms of peripheral neuropathy. Based on empirical evidence of its efficacy, GJG has been used to treat diabetic neuropathy [5, 6], and the clinical use of GJG for the treatment and prevention of CIPN is increasing [7, 8]. Although several studies have examined the effects of GJG on CIPN [8, 9], the active constituents of GJG responsible for efficacy in CIPN and the molecular mechanism of their pharmacological effect are not fully understood.

Previously, we compared the anti-allodynic effect of GJG and Hachimijiogan, a related formulation, using a PTX induced allodynia mouse model. Hachimijiogan is derived from GJG and lacks two crude drug ingredients, Plantaginis Semen and Achyranthis Radix. Interestingly, while GJG exhibited anti-allodynic effects, Hachimijiogan was devoid of this activity. These observations suggest that Plantaginis Semen and/or Achyranthis Radix may be involved in the anti-allodynic activity of GJG [10]. Follow-up study revealed that the administration of water extract of Plantaginis Semen (EPS, 0.3 g/kg, oral, daily) resulted in significant inhibition of PTX-induced mechanical allodynia. This effect of EPS was dose-dependent as a lower dose of EPS (0.1 g/kg, oral, daily) lacked significant anti-allodynic activity. In contrast, the administration of the water extract of Achyranthis Radix (EAR, 0.3–0.03 g/kg, oral, daily) did not inhibit the PTX-induced mechanical allodynia [11], suggesting the presence of active chemical constituents in Plantaginis Semen with anti-allodynic properties. In the present study, identification of anti-allodynic compounds in EPS using activity-guided separation of EPS and the evaluation of isolated compounds in PTX-induced allodynia mouse model was performed.

## Materials and methods

### Samples and isolation

The water extract of Plantaginis Semen (the seed of *Plantago asiatica*) was provided by Tsumura & Co (Lot No. 1049). The extract (170 g) was first dissolved in 8.5 L of water followed by addition of 12.75 L of methanol (MeOH) to make a 60% MeOH solution, which resulted in precipitation of polysaccharide sediment. After centrifugation at 4000 rpm for 15 min, supernatant was separated from sediment. The supernatant was diluted to 5% MeOH solution by addition of water and was subjected to Diaion HP-21 (Mitsubishi Chemical, Tokyo, Japan) column chromatography (100 mm i.d. × 600 mm, stepwise gradient elution with 0–100% MeOH) and six fractions were eluted: water eluent (3650 mg), 20% MeOH eluent (1099 mg), 40% MeOH eluent (2853 mg), 60% MeOH eluent (4800 mg), 80% MeOH eluent (852 mg), and 100% MeOH eluent (397 mg). With the help of Nippon Funmatsu Yakuhin Co., LTD (Osaka, Japan), scale-upped extraction from 8.1 kg of Plantaginis Semen and removal of polysaccharide sediment as described above yielded 380 g of processed extract (Lot No. 15D20). A portion (160 g) of the processed extract was subjected to Diaion HP-21 column chromatography as described above to yield 10.4 g of 20% MeOH eluent.

Octadecylsilyl medium-pressure liquid chromatography (ODS-MPLC) was performed (Biotage SNAP Ultra C18 120 g, 42 mm i.d. × 150 mm) on 8.8 g the of 20% MeOH fraction followed by elution with a gradient of 5–100% MeOH in water, to obtain seven fractions, 1A to 1G. Fraction 1C (1.39 g) was further separated by preparative C30 HPLC [Develosil C30-UG-5, 20 mm i.d. × 250 mm with 20 mm i.d. × 50 mm guard column; flow rate 10 mL/min; solvent A: water with 0.1% formic acid; solvent B: CH_3_CN; gradient elution 4% solvent B (0–10 min) and 4–30% solvent B (10–32 min), detection: UV at 210 nm] which resulted in isolation of compounds **4** (23.5 mg, t_*R*_ 12 min), **3** (13.8 mg, t_*R*_ 16.5 min), **1** (19.6 mg, t_*R*_ 20 min), and **2** (74.2 mg, t_*R*_ 24.5 min). Tap water was used for the extraction and HP-21 column chromatography, while purified water was used for the MPLC and preparative HPLC. These isolated compounds were identified by ^1^H-NMR (ECA-500II spectrometer, JEOL, Tokyo, Japan) and high-resolution electrospray ionization time-of-flight mass spectrometry (HR-ESI-TOFMS, Shimadzu, Kyoto, Japan). For compounds **1** and **2**, commercially available standards were also used for identification by LCMS.

In order to prepare the samples for animal experiments, the above mentioned methods using Diaion HP-21 column chromatography and ODS-MPLC were employed with slight modifications to obtain the fractions **crude 3** and **crude 4**. The content of **3** or **4** in **crude 3** and **crude 4** was estimated by quantitative ^1^H-NMR method (see Supplementary material). For preparation for **2**, in addition to the above mentioned methods, preparative C30 HPLC was employed with slight modifications.

### Animal experiments

#### Animals

Six-weeks old male C57BL/6NCr mice (Japan SLC, Shizuoka, Japan) were used in the mouse model of allodynia. Mice were housed under controlled temperature (21–23 °C), humidity (45–65%), and light (7:00 AM to 7:00 PM, 12-h light/dark cycle) conditions. Food and water were available ad libitum. This study was approved by the Committee for Animal Experiments at the University of Toyama and was performed in accordance with the guidelines for investigations of experimental pain in animals published by the International Association for the Study of Pain.

#### Drugs

PTX (Sigma, St. Louis, MO, USA) was dissolved in the vehicle [vehicle 1; physiological saline containing 10% Cremophor EL^®^ (Sigma) and 10% ethanol] and was administered intraperitoneally (5 mg/kg) at 0.1 mL/10 g of body weight. The dose of PTX was calculated based on the recommended clinical doses [12]. The fractions, purified compounds, and aucubin (Wako Pure Chemical Industries, Osaka, Japan) were dissolved in 5% solution of gum arabic in water (vehicle 2) and administered orally in a volume of 0.1 mL/10 g of body weight once daily, starting the day after PTX injection.

#### Behavioral experiments

Mechanical allodynia of the hind paw was assessed using a fine von Frey filament with a bending force of 0.69 mN (North Coast Medical Inc., Morgan Hill, CA, USA) [12]. The mice were placed individually in an acrylic cage (11 cm × 18 cm × 15 cm) with a wire mesh bottom. After an acclimation period of at least 30 min, the von Frey filament was pressed perpendicularly against the central part of the plantar hind paw of a freely-moving mouse and was held there for 1–3 s with the filament slightly buckled. Responses to the stimulus were ranked as follows: 0, no reaction; 1, lifting of the hind paw; and 2, licking and flinching of the hind paw. The stimulation of the same intensity was applied to each hind paw three times at intervals of several seconds, and the allodynia score (the total score of six tests) was expressed as a percentage of a maximum score of 12.

### Statistical analysis

Data were represented as the mean ± standard error of the mean (SEM). Statistical significance was determined using two-way repeated measures analysis of variance (ANOVA) and Bonferroni post hoc test for multiple comparisons. A *p* value of less than 0.05 was considered statistically significant.

## Results

**Fig. 1:**
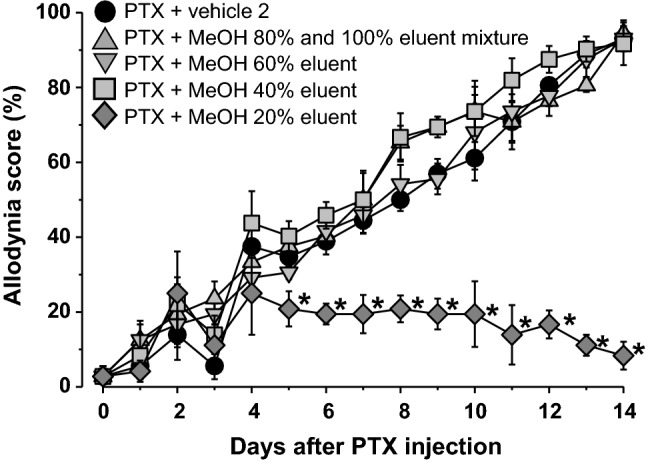
Effects of repetitive oral administration of the fractions separated by Diaion HP-21 column chromatography on PTX-induced mechanical allodynia. PTX (5 mg/kg) was injected intraperitoneally in mice. Fractions or vehicle 2 (5% gum arabic in water) were administered orally once daily starting the day after PTX injection. Data are presented as mean ± standard error of the mean (*N* = 6). *p < 0.05 vs. PTX + vehicle 2 (Bonferroni multiple comparisons)

**Fig. 2:**
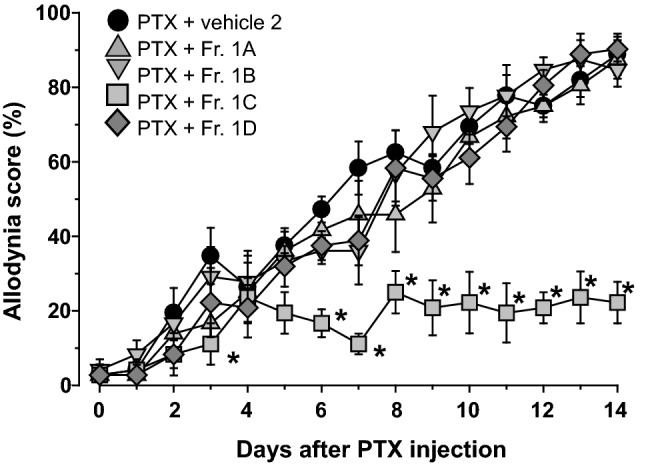
Effects of repetitive oral administration of the fractions (Fr. 1**A**–1**D**) separated by MPLC on PTX-induced mechanical allodynia. PTX (5 mg/kg) was injected intraperitoneally in mice. Fractions or vehicle 2 (5% gum arabic in water) were administered orally once daily starting the day after PTX injection. Data are presented as mean ± standard error of the mean (*N* = 6). *p < 0.05 vs. PTX + vehicle 2 (Bonferroni multiple comparisons)

**Fig. 3:**
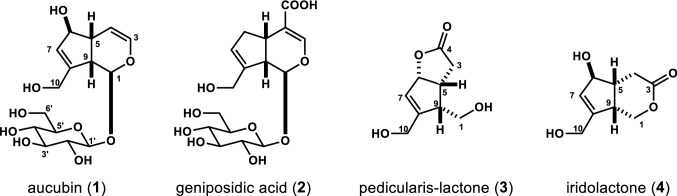
Structure of compounds **1**–**4**

In order to identify the anti-allodynic compounds in Plantaginis Semen, activity-guided separation of its hot water extract was conducted. After removal of polysaccharide sediment by precipitation using MeOH, the supernatant was subjected to Diaion HP-21 column chromatography to obtain six fractions (water eluent, 20%, 40%, 60%, 80%, and 100% MeOH eluent). The anti-allodynic effects of these fractions were evaluated in PTX-treated mice. A single administration of PTX (5 mg/kg, i.p.) induced mechanical allodynia. In this model, the allodynia score peaked on day 14 and almost renormalized by day 39 [11]. Therefore, we evaluated the effect of fractions and compounds on allodynia each day up to day 14 after PTX injection. The allodynia score for all fractions was the same as the vehicle 2 group, except for the 20% MeOH eluent (0.3 g/kg), which decreased the allodynia score significantly (Fig. 1). These results suggested the presence and enrichment of anti-allodynic compounds in 20% MeOH eluent fraction. Scale-up of the extraction process allowed ODS-MPLC separation of the 20% MeOH fraction to yield seven subfractions, 1A–1G. Among these seven fractions, only fraction 1C (0.15 g/kg) showed anti-allodynic activity (Fig. 2). The allodynia score for fractions 1E − 1G showed no significant difference from the vehicle 2 group (data not shown). From fraction 1C, four compounds (**1**–**4**) were purified by preparative C30 HPLC. These compounds were identified to be aucubin (**1**) [13], geniposidic acid (**2**) [14], pedicularis-lactone (**3**) [15], and iridolactone (**4**) [15], by means of spectroscopic analysis and by comparison of their MS and NMR data with those in the published literature (Fig. 3). To evaluate the anti-allodynic activity for these compounds in vivo, more than 300 mg of the samples were prepared as follows. Commercially available **1** was used, while **2** was purified using preparative C30 HPLC. Due to their low content in Plantaginis Semen, preparation of purified **3** and **4** for animal experiments was challenging. However, fractions **crude 3** and **crude 4**, containing these compounds, respectively, were prepared by ODS-MPLC and used for activity evaluation in mice. Using ^1^H-NMR, we determined that the principal compound in each fraction was **3** or **4**, respectively. Quantitative ^1^H-NMR showed that the estimated content of **3** in **crude 3** was 13.4 ± 0.2%, and that of **4** in **crude 4** was 8.4 ± 0.7%.

**Fig. 4:**
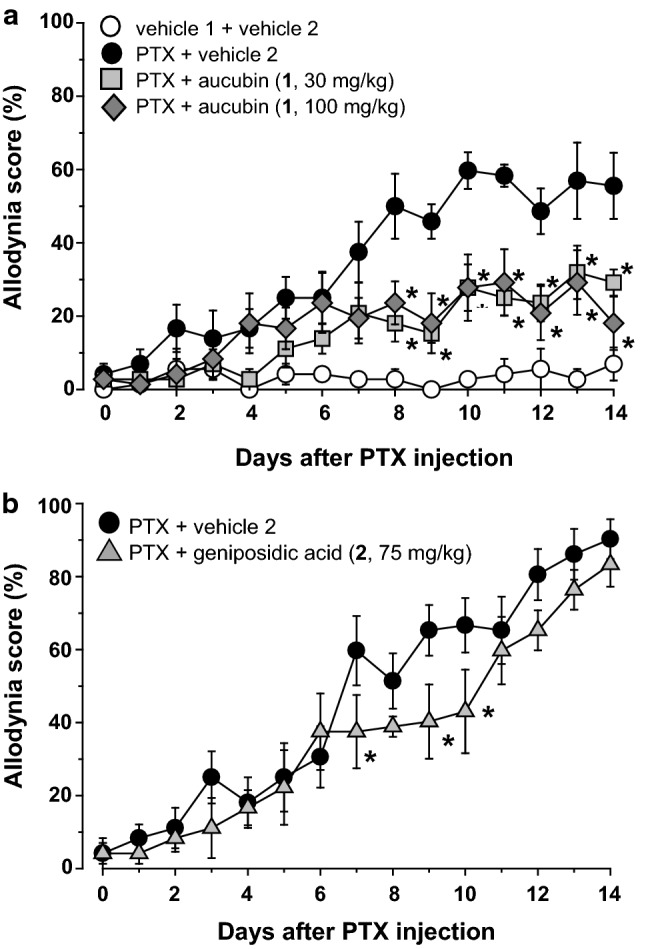
Effects of repetitive oral administration of aucubin (**1**) and geniposidic acid (**2**) on PTX-induced mechanical allodynia. PTX (5 mg/kg) or vehicle 1 (physiological saline containing 10% Cremophor EL^®^ (Sigma) and 10% ethanol) was injected intraperitoneally in mice. Aucubin (**1**, **a**), geniposidic acid (**2**, **b**), or vehicle 2 (5% gum arabic in water) was administered orally once daily from the day after PTX injection. Data are presented as mean ± standard error of the mean (*N* = 6). *p < 0.05 vs. PTX + vehicle 2 (Bonferroni multiple comparisons)

**Fig. 5:**
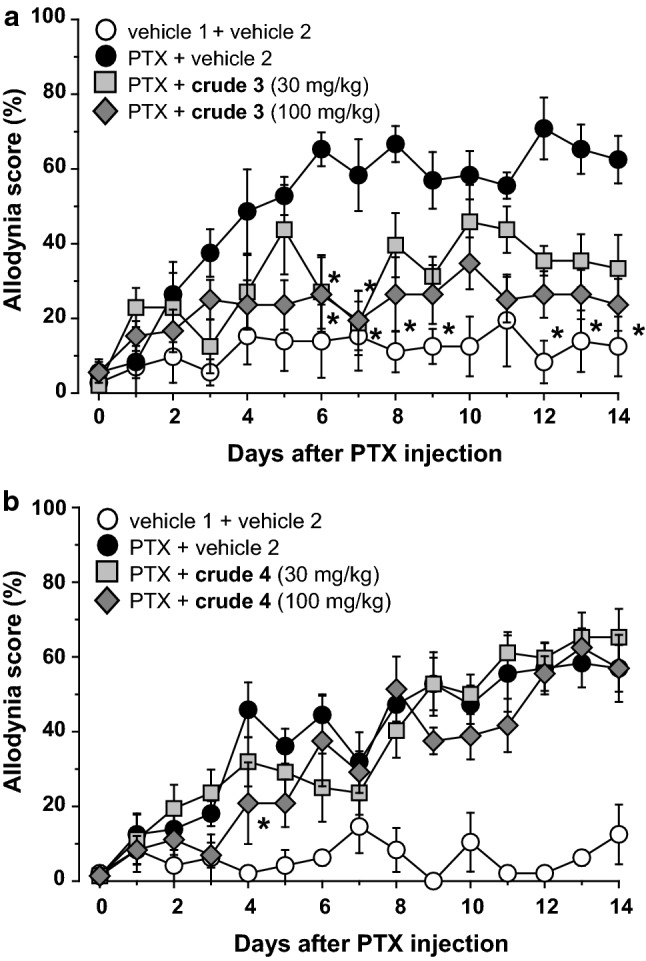
Effects of repetitive oral administration of **crude 3** or **crude 4** on PTX-induced mechanical allodynia. PTX (5 mg/kg) or vehicle 1 (physiological saline containing 10% Cremophor EL^®^ (Sigma) and 10% ethanol) was injected intraperitoneally in mice. **Crude 3** (**a**), **crude 4** (**b**) or vehicle 2 (5% gum arabic) were administered orally once daily starting the day after PTX injection. Data are presented as mean ± standard error of the mean (*N* = 4–6). *p < 0.05 vs. PTX + vehicle 2 (Bonferroni multiple comparisons)

Anti-allodynic effect of the isolated compounds was evaluated using a PTX-induced mechanical allodynia mouse model. Daily oral administration of aucubin (**1**, 100 mg/kg), starting the day after PTX injection, significantly inhibited mechanical allodynia, with effects observed from day 8 onwards. A lower dose of **1** (30 mg/kg) also retained the similar inhibition, evident after day 8 (Fig. 4a). Geniposidic acid (**2**, 75 mg/kg), as shown in Fig. 4b, significantly decreased the allodynia score on days 7, 9, and 10 compared to the vehicle 2 treated group. However, this effect was not sustained, and the score gradually increased in a manner similar to vehicle control, suggesting that **2** did not affect the mechanical allodynia induced by PTX. As mentioned above, the activities of pedicularis-lactone (**3**) and iridolactone (**4**) were evaluated using a fraction containing **3** as its major component (named as **crude 3**) and a fraction containing **4** as its major component (**crude 4**), respectively. Significant inhibition of mechanical allodynia was observed starting at day 6 upon oral administration of **crude 3** (100 mg/kg), an effect also observed for a lower dose of **crude 3** (30 mg/kg), although statistically insignificant (Fig. 5a). Oral administration of **crude 4** (30 and 100 mg/kg) exhibited no inhibition (Fig. 5b).

## Discussion

Previously we have reported that once daily oral administration of GJG (1.0 g/kg) [10] and EPS (0.3 g/kg) [11], an ingredient crude drug of GJG, inhibit PTX-induced mechanical allodynia in mice. These findings suggest that the anti-allodynic activity of GJG could be ascribed to Plantaginis Semen. In the present study, therefore, activity-guided separation of EPS was performed and led to identification of four iridoids (**1**–**4**). Aucubin (**1**) and geniposidic acid (**2**) have previously been known to be the major compounds in Plantaginis Semen [16]. The iridoids, pedicularis-lactone (**3**) and iridolactone (**4**), have been isolated from the roots of *Pedicularis chinensis* [15] (Oribanchaceae), the roots of *Scrophularia buergeriana* [17] (Scrophulariaceae), and the fruits of *Vitex rotundifolia* [18] (Lamiaceae). Recently, their isolation from Plantaginis Semen has been reported [19].

Oral administration of **1** (30 and 100 mg/kg) inhibited mechanical allodynia in the PTX-induced mouse model of allodynia. Due to challenges in isolation of pure compounds sufficient for animal experiments, **crude 3** and **crude 4** were evaluated in lieu of pure compounds **3** and **4**. Orally administered **crude 3** (100 mg/kg) inhibited mechanical allodynia, suggesting the potential anti-allodynic activity of **3**. On the other hand, **2** and **crude 4** did not affect the mechanical allodynia. Collectively, these results indicate **1** and **3** as the active anti-allodynic compounds in Plantaginis Semen and GJG. Although the effects of Plantaginis Semen extract on pain perception haven’t yet been reported, the analgesic effect of oral administration of iridoids, including **1** and **2,** was reported in a mouse model [20]. Thus, it is plausible that the analgesic activity of 1 may have a role in its anti-allodynic effect.

Although the quantitative levels of **3** in **crude 3** was not high, it is the major compound of **crude 3** as suggested by ^1^H-NMR spectral data. Since tap water was used in the isolation process, it is likely that other constituents of **crude 3** are water soluble compounds such as minerals and sugars. Minerals are undetectable in the ^1^H-NMR as they lack a proton in the molecule. ^1^H-NMR signals, likely originating from sugars, ranging between 3 and 4 ppm were detected. Based on our current knowledge of **crude 3** constituents, we postulate that **3** is one of the active anti-allodynic compounds in Plantaginis Semen and GJG. Evaluation of purified compound **3** in animal experiments will be needed to obtain additional confirmation. The purification of **3** from Plantaginis Semen presented significant challenges due to its low levels. Therefore, the purification of **3** from other crude drugs is in currently being pursued.

Xue et al. report the pharmacokinetics of aucubin intravenous administration as follows: plasma aucubin level reaches peak concentration about 0.08 h after administration (*Tmax*) and the elimination half-life (*T*_*1/2*_) is 1.07 h. And also, they indicate that pharmacokinetic property of aucubin in TCM extract may change and may be inhibited depending on the other ingredients [21]. Regarding oral administration of aucubin, Xu et al. report that *Tmax* was about 1 h and *T*_*1/2*_ is 7.4 h [22]. In our mouse experiment, evaluation of allodynia (von Frey test) was conducted after approximately 24 h of administration. The plasma level of aucubin may not be high at the time point of the von Frey test. As shown in Ref. [11], Andoh et al. report that single intraperitoneal injection of aucubin does not inhibit PTX-induced mechanical allodynia, suggesting that aucubin does not have acute anti-allodynic activity. Taken together, it is suggested that the alteration of plasma level of aucubin and inhibition of PTX-induced allodynia do not have direct correlation. Anti-allodynic activity by repetitive administration of aucubin may be due to organic protection, such as the protection of demyelination [23].

In our previous study [11], we have reported that repetitive intraperitoneal injection of aucubin (**1**, 50 mg/kg) inhibits mechanical allodynia induced by PTX in mice. Additionally, intraperitoneally administered **2** (50 mg/kg) and catalpol (50 mg/kg), related iridoid glucosides of aucubin, did not inhibit allodynia in mice. In this study, we validated the anti-allodynic effect of **1** by oral administration. In our preliminary experiment, we detected **1** in the plasma sample of mice after oral administration of EPS (1 g/kg), suggesting that orally administered **1** was absorbed into the blood to exhibit anti-allodynic effects (data not shown). In a study on a mouse model of breast cancer, repetitive oral administration of GJG (1 g/kg) inhibited the PTX-induced allodynia without affecting the anti-cancer activity of PTX [24]. We speculate that **1** and **3**, the active anti-allodynic constituents of Plantaginis Semen will not interfere with chemotherapy. However, further studies are needed to confirm this assumption.

There is evidence to suggest the underlying mechanism of anti-allodynic activity of **1**. We reported that intraperitoneal injection of **1** inhibited firing induced by mechanical stimuli in spinal dorsal horn neurons in PTX-treated mice. Furthermore, an endoplasmic reticulum (ER) stress inhibition mechanism is suggested by the inhibitory effect of **1** on the PTX-induced expression of CCAAT/enhancer-binding protein homologous protein, a protein marker of ER stress, in the mouse sciatic nerve and LY-PPB6 cells (a rat Schwann cell line) [23]. Demyelination associated with chronic pain is induced by PTX in peripheral nerves [25, 26], because PTX causes damage in the Schwann cells [25], which form myelin sheaths, via ER stress [27, 28]. As mentioned above, we detected aucubin in the plasma sample of mice after oral administration of EPS. Therefore, we expect that oral administration of **1** may produce anti-allodynic effects via the same mechanism as intraperitoneal administration.

Recently case reports of association between the use of Kampo formulas, especially containing Gardeniae Fructus (GF), and mesenteric phlebosclerosis are increasing [29]. Among several iridoid glycosides reported from GF, the responsible compound for MP in GF is suggested to be geniposide [30, 31]. On the other hand, although several iridoid glycosides from Plantaginis semen had been reported, to the best of our knowledge, the isolation of geniposide from Plantaginis Semen (the seed of *Plantago asiatica*) had not been reported [32, 33]. The contents of major iridoid glycosides in Plantaginis Semen had been reported as follows: catalpol, 179.6 μg/g; aucubin, 104.2 μg/g; and geniposidic acid, 7552 μg/g [34]. The Japanese Pharmacopoeia, Seventeenth Edition (JP17) prescribes that GF contains not less than 3.0% of geniposide. Thus, the content of iridoid glycosides in Plantaginis Semen could be considered to be lower than the geniposide content in GF.

Normally the period of anti-cancer agent treatment is less than one year, in cancer chemotherapy. Consequently, the period of GJG treatment for CIPN is also less than one year. According to Nagata’s report [29], the average period of GF administration in MP case patients was 11.1 years (4.0–15.9 years). In our 14-day mice experiment the dark-purple change of mucosal surface in the colon, a typical finding of MP, was not observed when administered the fraction or compound. In the present, there is not enough information on MP and long period administration of GJG. Iridoid glycoside content is low in GJG, however, the long period of administration should be noted on the adverse effects, including MP.

## Conclusion

Our study helps to clarify the active constituents of GJG and its ingredient crude drug, Plantaginis Semen, which possess anti-allodynic activity. Activity-guided separation of the hot water extract of Plantaginis Semen led to the isolation of four iridoids (**1**–**4**). In addition to aucubin (**1**), repetitive oral administration of crude pedicularis-lactone (**3**) showed anti-allodynic action. Collectively, this study  suggest that **1** and **3** can be some of the active anti-allodynic compounds in Plantaginis Semen and GJG. Aucubin (**1**) and pedicularis-lactone (**3**) have the potential for therapeutic application in the clinical management of CIPN.

## Electronic supplementary material

Below is the link to the electronic supplementary material.

Supplementary material 1 (DOCX 146 kb)
