# Correction to: Search of anti-allodynic compounds from Plantaginis Semen, a crude drug ingredient of Kampo formula “Goshajinkigan”

**DOI:** 10.1007/s11418-020-01404-x

**Published:** 2020-04-10

**Authors:** Kazufumi Toume, Zhiyan Hou, Huanhuan Yu, Mitsuru Kato, Miki Maesaka, Yanjing Bai, Shiho Hanazawa, Yuewei Ge, Tsugunobu Andoh, Katsuko Komatsu

**Affiliations:** 1 Division of Pharmacognosy, Institute of Natural Medicine, University of Toyama, 2630 Sugitani, Toyama, Toyama 930-0194 Japan; 2 Department of Applied Pharmacology, Graduate School of Medicine and Pharmaceutical Sciences, University of Toyama, 2630 Sugitani, Toyama, Toyama 930-0194 Japan

## Correction to: Journal of Natural Medicines (2019) 73:761–768 https://doi.org/10.1007/s11418-019-01327-2

The article Search of anti-allodynic compounds from Plantaginis Semen, a crude drug ingredient of Kampo formula “Goshajinkigan”, written by Kazufumi Toume, Zhiyan Hou, Huanhuan Yu, Mitsuru Kato, Miki Maesaka, Yanjing Bai, Shiho Hanazawa, Yuewei Ge, Tsugunobu Andoh and Katsuko Komatsu was originally published Online First without Open Access. After publication in volume 73 issue 4, page 761–768 the author decided to opt for Open Choice and to make the article an Open Access publication. Therefore, the copyright of the article has been changed to © The Author(s) 2020 and the article is forthwith distributed under the terms of the Creative Commons Attribution 4.0 International License (https://creativecommons.org/licenses/by/4.0/), which permits use, duplication, adaptation, distribution and reproduction in any medium or format, as long as you give appropriate credit to the original author(s) and the source, provide a link to the Creative Commons license, and indicate if changes were made.

The original article was updated.

