# Supplementary material for: Search of anti-allodynic compounds from Plantaginis Semen, a crude drug ingredient of Kampo formula “Goshajinkigan”
Source: J Nat Med. 2019 Jun 12;73(4):761–8. doi: 10.1007/s11418-019-01327-2 (PMC7176603; doi:10.1007/s11418-019-01327-2)
Supplement: Supplementary file 1 — Supplementary material 1 (DOCX 146 kb) [file 11418_2019_1327_MOESM1_ESM.docx]

**Supplementary Material**

Search of anti-allodynic compounds from Plantaginis Semen, a crude drug ingredient of Kampo formula “Goshajinkigan”

Kazufumi Toume^1,^*, Zhiyan Hou^1^, Huanhuan Yu^1^, Mitsuru Kato^2^, Miki Maesaka^2^, Yanjing Bai^1^,
Shiho Hanazawa^1^,Yuewei Ge^1^, Tsugunobu Andoh^2^, Katsuko Komatsu^1,^*

^1^ Division of Pharmacognosy, Institute of Natural Medicine, University of Toyama, 2630 Sugitani, Toyama 930-0194, Toyama, Japan

^2^ Department of Applied Pharmacology, Graduate School of Medicine and Pharmaceutical Sciences, University of Toyama, 2630 Sugitani, Toyama 930-0194, Toyama, Japan

*Corresponding author

Kazufumi Toume
E-mail address: toume@inm.u-toyama.ac.jp, Phone number +81-76-434-7609

Katsuko Komatsu
E-mail address: katsukok@inm.u-toyama.ac.jp, Phone number +81-76-434-7601

**Contents**

1. General Experimental Procedures S3

2. Identification of isolated compounds (**1**-**4**) S3

3. Estimation of content of compounds **3** and **4** in the fractions **crude 3** and **crude 4**, respectively, by quantitative ^1^H-NMR (qHNMR) S5

**1. General Experimental Procedures**

The following instruments were used in the present study: a P-2100 digital polarimeter (JASCO) for optical rotations; an ECA-500II spectrometer (JEOL) for NMR spectroscopy (solvent chemical shifts were used as the internal standard); and a Shimadzu LCMS system (Shimadzu) consisting of DGU-20A5 on-line degasser, LC-20AD pumps (2 units), SIL-20A autosampler, CTO-20A column oven, SPD-M20A PDA detector, LCMS-IT-TOF hybrid ion trap time-of-flight (IT-TOF) mass spectrometer equipped with an ESI (electrospray ionization) interface was used and chromatographic data were collected and processed using LCMS Solution software (version 3.81, Shimadzu). TFA-Na (sodium trifluoroacetate) solution was used as the standard sample for turning and calibration (ion trap and TOF analyzer). Data was acquired using following parameters: detector voltage, 1.80 kV; probe voltage, +4.5 kV (positive mode) or -3.5 kV (negative mode); nebulizing gas flow, 1.5 L/min.; drying gas pressure, 100 kPa; temperature for CDL (curved desolvation line) and heat block, 200°C; ion accumulation time, 30 msec.; scanning range, m/z 100-1000. Following LC-MS condition was used. Column: Develosil C30-UG-5 (5 μm, 2.0×150 mm), flow rate: 0.2 mL/min,

Solvent system: A; water with 0.1% formic acid, B; MeCN with 0.1% formic acid,

Gradient conditions: 4% B (0-2min), 4-30% B (2-6 min), 30%-40% B (6-20 min).

MS range: *m/z* 100-2000 at negative mode.

**2. Identification of isolated compounds (1-4)**

Isolated compounds (**1**-**4**) were identified to be aucubin (**1**)^S1^, geniposidic acid (**2**) ^S2^, pedicularis-lactone (**3**) ^S3^, and iridolactone (**4**) ^S3^ by means of spectroscopic analysis and comparison of their MS and ^1^H-NMR data (Table S1) with those in the literature.

In LC/MS analysis, the retention time (*t*_R_) and *m/z* values of compounds **1** and **2** were identical with those of commercially available standard aucubin (Wako) and geniposidic acid (Wako).

The retention time (*t*_R_) and *m/z* values of **1**-**4** and [α]_D_ values of **3** and **4** are shown in below.

aucubin (**1**): *t*_R_ 5.18 min, *m/z* 369.1137 [M+Na]^+^ (calcd for 369.1156, Δ-1.9 mmu), *m/z* 391.1249 [M+HCOO]^-^ (calcd for 391.1246, Δ+0.3 mmu)

geniposidic acid (**2**): *t*_R_ 8.68 min, *m/z* 373.1143 [M-H]^-^ (calcd for 373.1140, Δ+0.3 mmu), *m/z* 747.2369 [2M-H]^-^ (calcd for 747.2353, Δ+1.6 mmu)

pedicularis-lactone (**3**): *t*_R_ 4.31 min, *m/z* 185.0797 [M+H]^+^ (calcd for 185.0808, Δ-1.1 mmu), *m/z* 207.0604 [M+Na]^+^ (calcd for 207.0628, Δ+2.4 mmu), [α] +33.4 (*c* 0.19, MeOH)

iridolactone (**4**): *t*_R_ 3.14 min, *m/z* 185.0812 [M+H]^+^ (calcd for 185.0808, Δ+0.4 mmu), *m/z* 207.0629 [M+Na]^+^ (calcd for 207.0628, Δ+0.1 mmu), [α] -12.8 (*c* 0.26, MeOH)

**Table S1** ^1^H-NMR spectroscopic data for **1**-**4**

|  | aucubin (**1**) | geniposidic acid (**2**) | pedicularis-lactone (**3**) | | iridolactone (**4**) | |
| --- | --- | --- | --- | --- | --- | --- |
| position | δ_H_ in D_2_O (*J* in Hz) | δ_H_ in D_2_O (*J* in Hz) | δ_H_ in DMSO-d_6_ (*J* in Hz) | δ_H_ in D_2_O (*J* in Hz) | δ_H_ in DMSO-d_6_ (*J* in Hz) | δ_H_ in D_2_O (*J* in Hz) |
| 1 | 5.12 d (4.9) 1H | 5.19 d (6.7) 1H | 3.44 dd (10.5, 6.5) 1H | 3.58 dd (11.6, 3.7) 1H | 4.15 dd (11.5, 3.7) 1H | 4.22 dd (12.0, 3.4) 1H |
|  |  |  | 3.54 dd (10.5, 3.8) 1H | 3.61 dd (11.6, 4.3) 1H | 4.29 dd (11.5, 4.0) 1H | 4.29 dd (12.0, 4.0) 1H |
| 3 | 6.14 d (6.1) 1H | 7.44 s,1H |  |  |  |  |
| 4 | 4.96 dd (3.1, 6.1) 1H |  | 2.54 dd (18.4, 9.9) 1H | 2.56 dd (18.9, 3.7) 1H | 2.40 dd (14.9, 4.6) 1H | 2.44 dd (15.5, 3.4) 1H |
|  |  |  | 2.59 dd (18.4, 5.4) 1H | 5.66 dd (18.9, 10.4) 1H | 2.77 dd (14.9, 7.5) 1H | 2.78 dd (15.5, 8.0) 1H |
| 5 | 2.63 m, 1H | 3.08 dd (7.9, 14.7) 1H | 3.17 m, 1H | 3.23 m, 1H | 2.47 m, 1H | 2.64 m, 1H |
| 6 | 4.37 br s, 1H | 2.04 dd (6.1, 16.5) 1H | 5.33 d (7.6), 1H | 5.41 d(7.3) 1H | 4.23 br s, 1H | 4.36 br s, 1H |
|  |  | 2.68 dd (8.5, 16.5) 1H |  |  |  |  |
| 7 | 5.70 br s, 1H | 5.74 s, 1H | 5.71 s, 1H | 5.79 s, 1H | 5.58 s, 1H | 5.68 s, 1H |
| 9 | 2.99 t (5.5) 1H | 2.75 dd (6.7, 7.4) 1H | 2.83 br s, 1H | 2.89 br s, 1H | 3.12 br s, 1H | 3.29 m, 1H |
| 10 | 4.11 d (15.3) 1H | 4.11 d (14.0) 1H | 3.97 d (15.3) 1H | 4.08 d (15.3) 1H | 3.95 d (14.9) 1H | 4.04 d (15.2) 1H |
|  | 4.19 d (15.3) 1H | 4.17 d (14.0) 1H | 4.07 d (15.3) 1H | 4.15 d (15.3) 1H | 4.00 d (14.9) 1H | 4.08 d (15.2) 1H |
|  |  |  | 4.61 br s, 1H [1-OH] |  |  |  |
|  |  |  | 4.91 br s, 1H [10-OH] |  |  |  |
| 1’ | 4.63 m*, 1H | 4.70 d (8.0) 1H |  |  |  |  |
| 6’ | 3.57 dd (12.2, 5.5) 1H | 3.60 dd (12.1, 5.1) 1H |  |  |  |  |
|  | 3.76 dd (12.2, 1.2) 1H | 3.77 dd (12.1, 2.0) 1H |  |  |  |  |
| 2’-5’ | 3.16-3.38 m, 4H | 3.18-3.40 m, 4H |  |  |  |  |

* signal was overlapped with D_2_O

**3. Estimation of content of compounds 3 and 4 in the fractions crude 3 and crude 4, respectively, by quantitative ^1^H NMR (qHNMR)** ^S4,S5^**.**

**Experimental**

All the samples for qHNMR were weighted by ultra-microbalance Cubis MSA2.7S (Sartorius, Göttingen, Germany) (readability: 0.0001 mg). Sodium 3-(trimethylsilyl)-1-propane-1,1,2,2,3,3-*d*_6_-sulfonate (DSS-*d*_6_, Code No. 044-31671, Lot. EPL6585, Wako, Osaka, Japan) was used as an internal standard and D_2_O (Wako) was used as solvent for qHNMR. Following optimized sequence parameters were used for qHNMR: pulse angle, 90°; probe temperature, 25°C; spinning, off; number of scans, 8; spectral range, 20 ppm (-2 to 18 ppm); acquisition time, 4 s; relaxation delay, 60 s; pulse angle, 90°; pulse width, 6.4 μs; and ^13^C decoupling, multi-pulse decoupling with phase and frequency switching (MPF-8). The integral value of signal calculated by the software Delta 5.1.3 (JEOL) was used for the quantitative analysis. The chemical shift of all data was referenced to the residual HDO resonance at δ_H_ 4.65 ppm.

Sample solution for qHNMR was prepared as follows. Dried sample (5 mg) was accurately weighted and dissolved in 1 mL of internal standard solution. The aliquot (0.7 mL) of the solution was placed in a 5 mm NMR tube (Wako) with a height of 4 cm and subjected to qHNMR measurement. All the experiments were performed in triplicate.

The content assessment of pedicularis-lactone (**3**, C_9_H_12_O_4_) in **crude 3** was conducted using the H-7 signal at δ_H_ 5.79 ppm for 1H (Figure S1), while the content assessment of iridolactone (**4**, C_9_H_12_O_4_) in **crude 4** was conducted using the H-7 signal at δ_H_ 5.68 ppm for 1H (Figure S2). In these experiments, the final concentration of DSS-*d*_6_ (C_6_H_9_D_6_O_3_SSiNa) internal standard solution was 0.452 mg/mL and the purity of DSS-*d*_6_ was 92.3%. Content of **3** and **4** were calculated by the following equation:

$\mathrm{Content}_{s} (\%)=\frac{S_{s}}{S_{DSS}}\times\frac{N_{DSS}}{N_{s}}\times\frac{M_{s}}{M_{DSS}}\times\frac{C_{DSS}}{C_{s}}\times P_{DSS}\times100$

where S*_s_* and S*_DSS_* are the signal integral values of sample and DSS-*d*_6_, N*_s_* (= 1) and N*_DSS_* (= 9) are the number of protons of signal sample and DSS-*d*_6_, M*_s_* (184.19) and M*_DSS_* (244.35) are the molecular weight of sample and DSS-*d*_6_, C*_s_* and C*_DSS_* are the concentrations (mg/mL) of sample and DSS-*d*_6_ solution, P*_DSS_* is the purity of the DSS (92.3%), respectively.

**Results**


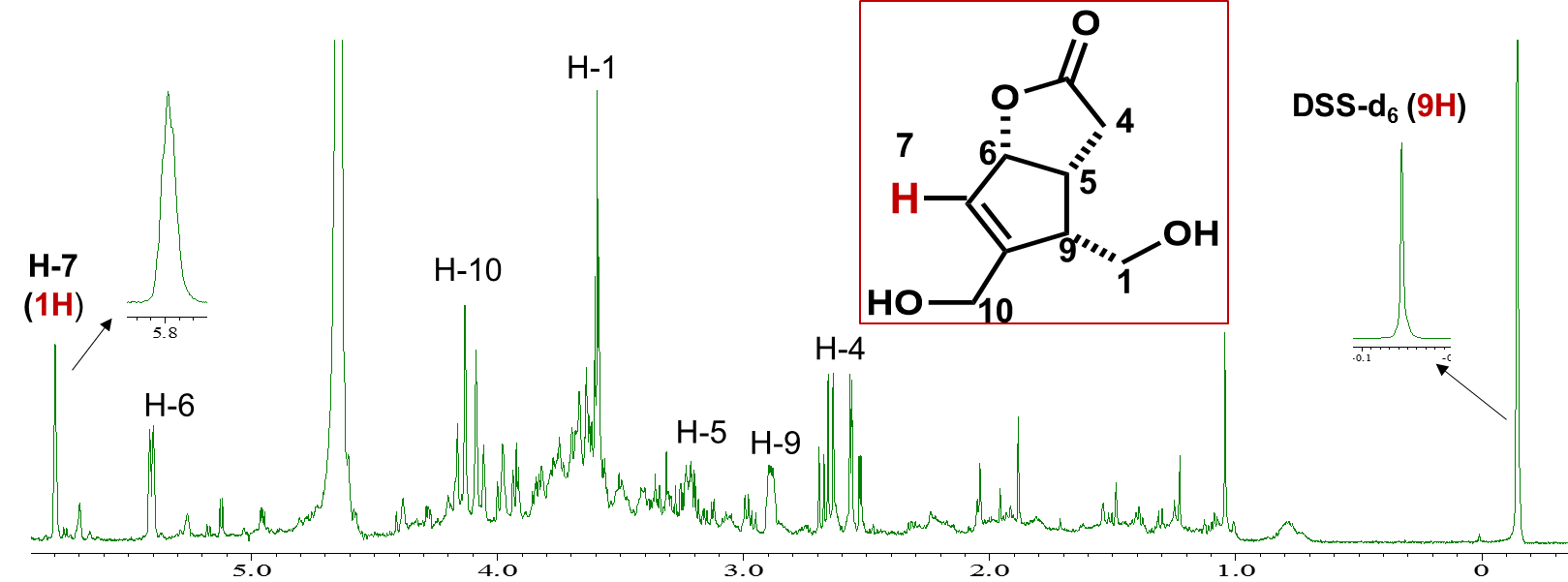

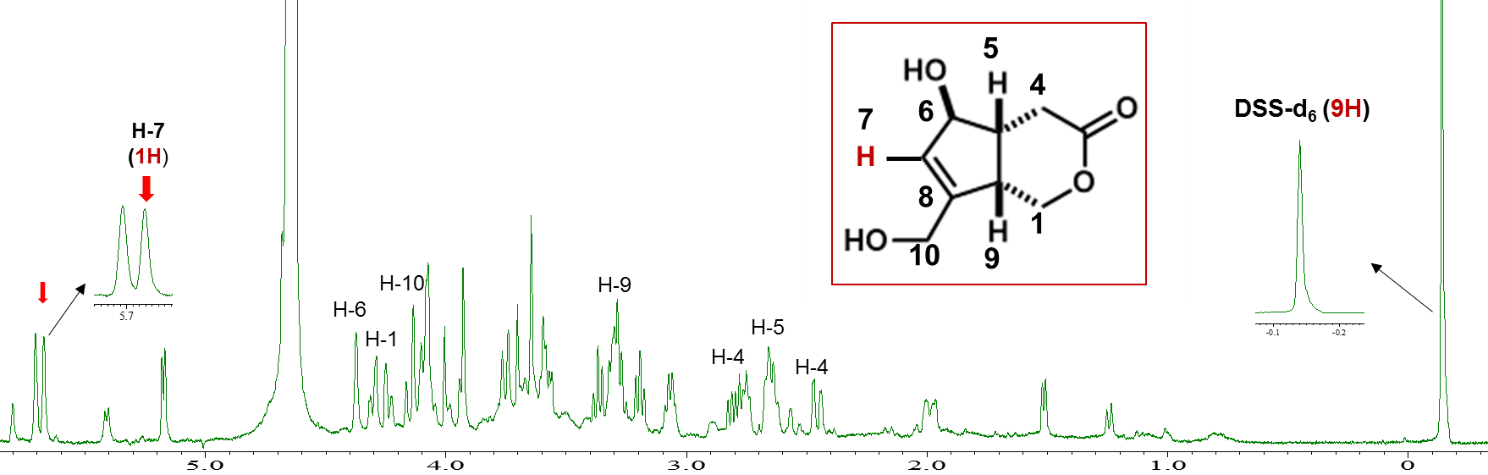
The content of pedicularis-lactone (**3**, C_9_H_12_O_4_) in **crude 3** was estimated using comparison of integral value of the H-7 signal at δ_H_ 5.71 ppm with those of DSS-*d*_6_ at δ_H_ -0.14 ppm (**Fig. S1**). As a result, the content of **3** in **crude 3** was estimated to be 13.4±0.2%. The content of pedicularis-lactone (**4**, C_9_H_12_O_4_) in **crude 4** was estimated using comparison of integral value of the H-7 signal at δ_H_ 5.58 ppm with those of DSS-*d*_6_ at δ_H_ -0.14 ppm (**Fig. S2**). As a result, the content of **4** in **crude 4** was estimated to be 8.4±0.7%.

**Fig. S1** ^1^H-NMR spectrum of **crude 3** and DSS-*d*_6_ in D_2_O

**Fig. S2** ^1^H-NMR spectrum of **crude 4** and DSS-*d*_6_ in D_2_O

**References**

S1. Davini, E.; Iavarone, C.; Trogolo, C.; Aureli, P.; Pasolini, B., The quantitative isolation and antimicrobial activity of the aglycon of aucubin. *Phytochemistry* **1986**, 25, 2420-2.

S2. Toda, S.; Miyase, T.; Arichi, H.; Tanizawa, H.; Takino, Y., Natural antioxidants. II. Antioxidative components isolated from seeds of Plantago asiatica Linne. *Chem. Pharm. Bull.* **1985**, 33, 1270-3.

S3. Li, Y.; Changzeng, W.; Zhongjian, J., Iridoids in roots of Pedicularis chinensis. *Phytochemistry* **1995**, 40, 491-494.

S4. Ohtsuki, T.; Sato, K.; Sugimoto, N.; Akiyama, H.; Kawamura, Y., Absolute quantification for benzoic acid in processed foods using quantitative proton nuclear magnetic resonance spectroscopy. *Talanta* **2012**, 99, 342-348.

S5. Tanaka, R.; Inagaki, R.; Sugimoto, N.; Akiyama, H.; Nagatsu, A., Application of a quantitative 1H-NMR (1H-qNMR) method for the determination of geniposidic acid and acteoside in Plantaginis semen. *J. Nat. Med.* **2017**, 71, 315-320.
